# Author Correction: A role for Biofoundries in rapid development and validation of automated SARS-CoV-2 clinical diagnostics

**DOI:** 10.1038/s41467-020-18720-1

**Published:** 2020-09-17

**Authors:** Michael A. Crone, Miles Priestman, Marta Ciechonska, Kirsten Jensen, David J. Sharp, Arthi Anand, Paul Randell, Marko Storch, Paul S. Freemont

**Affiliations:** 1 London Biofoundry, Imperial College Translation and Innovation Hub, White City Campus, 80 Wood Lane, London, W12 0BZ UK; 2 Section of Structural and Synthetic Biology, Department of Infectious Disease, Imperial College London, London, SW7 2AZ UK; 3 UK Dementia Research Institute Centre for Care Research and Technology, Imperial College London, London, UK; 4 Department of Brain Sciences, Imperial College London, Hammersmith Hospital, Du Cane Road, London, W12 0NN UK; 5 Histocompatibility and Immunogenetics Laboratories, Department of Infection and Immunity, North West London Pathology, London, UK; 6 Imperial College Healthcare NHS Trust, Hammersmith Hospital, Du Cane Road, London, W12 0HS UK; 7 Department of Infection and Immunity, North West London Pathology, London, UK; 8 Imperial College Healthcare NHS Trust, Charing Cross Hospital, Fulham Palace Road, Hammersmith, London, W6 8RF UK

**Keywords:** High-throughput screening, Assay systems, Synthetic biology, Infectious diseases

Correction to: *Nature Communications* 10.1038/s41467-020-18130-3, published online 08 September 2020.

The original version of this manuscript did not include a link to the RNA Extraction Procotol scripts that are publically available at Zenodo. The scripts can be found at the following URL: 10.5281/zenodo.4021454

In addition, the original version of this Article omitted a declaration from the Competing Interests statement, which should have included the following: “M. Priestman and M. Ciechonska are co-founders of Salient Labs. All other authors declare no competing interests.”

This has now been corrected in both the PDF and HTML versions of the Article.

