# Supplementary material for: A role for Biofoundries in rapid development and validation of automated SARS-CoV-2 clinical diagnostics
Source: Nat Commun. 2020 Sep 8;11:4464. doi: 10.1038/s41467-020-18130-3 (PMC7479142; doi:10.1038/s41467-020-18130-3)
Supplement: Supplementary file 1 — Supplementray Information [file 41467_2020_18130_MOESM1_ESM.pdf]

Supplementary Information – A new role for Biofoundries in rapid development and validation of automated SARS-CoV-2 clinical diagnostics

Crone et al.

Supplementary Figures

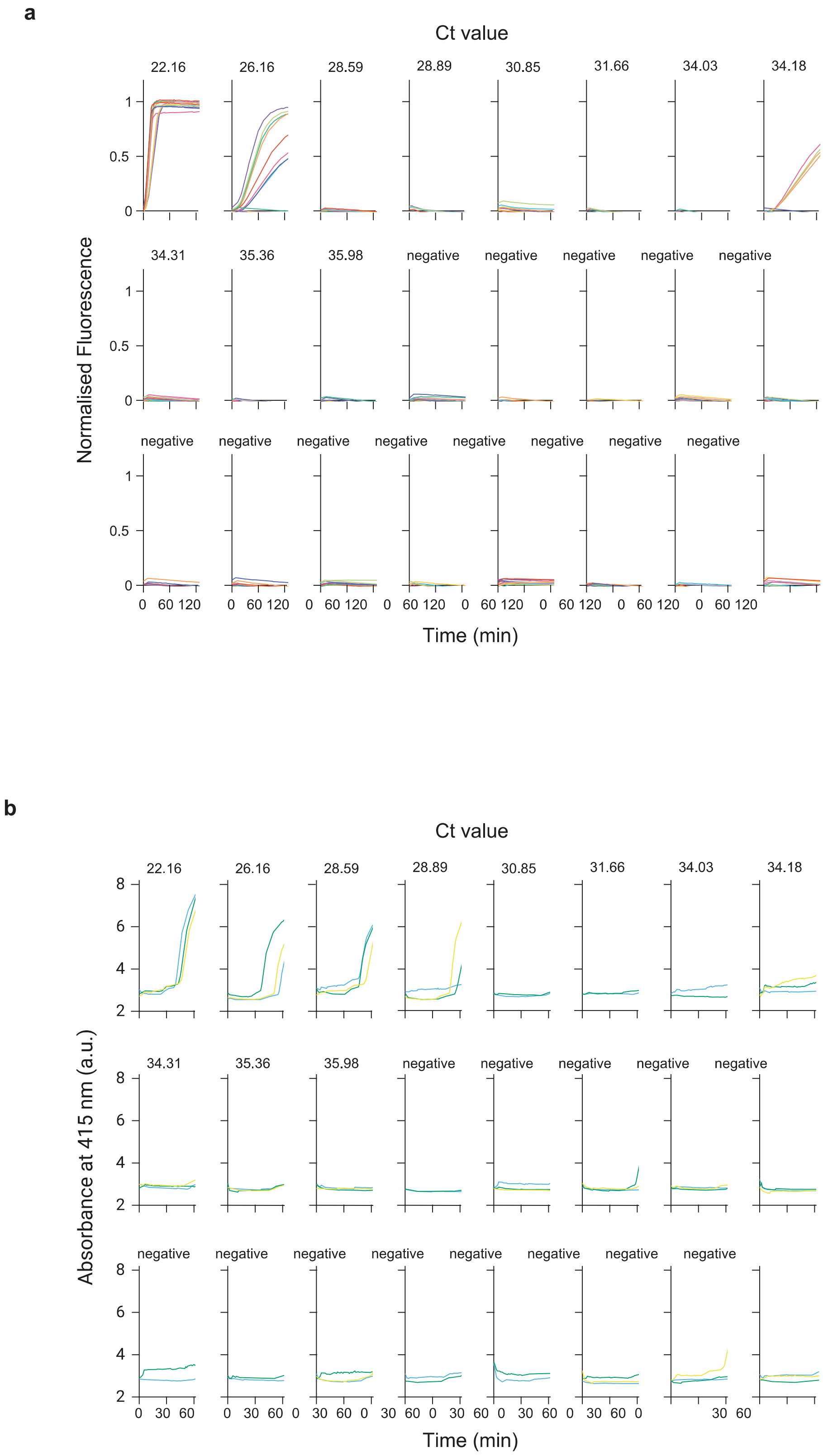

Supplementary Figure 1: Patient Sample Validation for LAMP and RPA-CRISPR Assays. (a) Individual traces for the RPA-CRISPR patient assays for 24 patient samples. **(b)** Individual traces for the colorimetric LAMP patient assays for 24 patient samples.

Supplementary Tables

Supplementary Table 1: Patient Sample Validation for LAMP and RPA-CRISPR Assays.

		Copies per reaction			
**Sample**	**RT-qPCR (ct value)**	**PCR**	**LAMP**	**RPA-CRISPR**	**Viral copies/mL viral transport media**
1	22.16	6409.91	2563.96	3204.95	320495.36
2	26.16	422.20	168.88	211.10	21109.93
3	28.59	80.88	32.35	40.44	4044.14
4	28.89	65.96	26.38	32.98	3297.81
5	30.85	17.39	6.96	8.70	869.70
6	31.66	10.03	4.01	5.01	501.36
7	34.03	2.00	0.80	1.00	100.05
8	34.18	1.81	0.72	0.90	90.35
9	34.31	1.65	0.66	0.83	82.70
10	35.36	0.81	0.32	0.40	40.50
11	35.98	0.53	0.21	0.27	26.56
12					
13					
14					
15					
16					
17					
18					
19					
20					
21					
22					
23					
24					

Copies per reaction are calculated using the line of best fit from the standard curve in Figure 2b ($y = -3.386x + 35.06$) and differ based on input volume between RT-qPCR (5 µL), LAMP (2 µL) and RPA-CRISPR (2.5 µL). The number of viral copies per millilitre of transport media is assuming 100% extraction efficiency with a sample volume of 200 µL and elution volume of 50 µL. Blocks highlighted in yellow were detected in the LAMP or RPA-CRISPR assays.

Supplementary Table 2: Summary of available diagnostic methodologies for high-throughput testing.

Assay	Commercial Reagents	Limit of Detection (gene)	Time to Result	HT Techniques	Advantages	Disadvantages
RT-qPCR (this work)	NEB (E3005L), ThermoFisher (A15299 or 4444432)	1 copy per reaction (N)	150 minutes	Automated RNA extraction, Echo setup	Highest sensitivity	Thermocycling
RT-LAMP (this work)	NEB (M1800L)	~30 copies per reaction (N)	100 minutes	Automated RNA extraction, Echo setup	Isothermal amplification	Potential non-specific amplification<br><br>No multiplexing in a single reaction
RT-PCR-CRISPR (this work)	RT-PCR: NEB (E3005L)<br><br>LwCas13a: In-house	~2.5 copies per reaction (N)	170 minutes	Automated RNA extraction, Echo setup	Plate reader instead of qPCR results in higher throughput	Thermocycling<br><br>More complicated setup
RPA-CRISPR (this work)	RPA: TwistDx (TALQBAS01)<br><br>LwCas13a: In-house	~200 copies per reaction (ORF1ab)	150-210 minutes	Automated RNA extraction, Echo setup	Higher throughput than RTqPCR	Not as sensitive as alternatives<br><br>More complicated assay setup
LAMP-CRISPR (IDT/Sherlock Biosciences)	IDT/Sherlock Biosciences (10006968)	360 copies per reaction (ORF1ab)<br><br>72 copies per reaction (N)	120 minutes	Multichannel setup	Short time to result	Very complicated assay setup<br><br>Low throughput (190 per shift per operator)

Supplementary Table 3: Primer and Probe Sequences for qPCR/RT-ddPCR

Figure	Name	Forward Primer	Reverse Primer	Probe
1d, 2a, 2b, 2c, 4b, 5b, 5c	2019-nCoV_N1	GACCCCAAAATCAG CGAAAT	TCTGGTTACTGCCAGTTGA ATCTG	FAM - ACCCCGCATTACGTTTGGTGGACC - BHQ1
2c	2019-nCoV_N2	TTACAAACATTGGCC GCAAA	GCGCGACATTCCGAAGAA	FAM - ACAATTTGCCCCCAGCGCTTCAG - BHQ1
2c	2019-nCoV_N3	GGGAGCCTTGAATA C ACCAAAA	TGTAGCACGATTGCAGCA TTG	FAM - AYCACATTGGCACCCGCAATCCTG - BHQ1

Supplementary Table 4: Primers and oligonucleotides for CRISPR-Cas13a reactions

Figure	Name	Forward primer/oligonucleotide	Reverse primer/oligonucleotide
3b, 4c	2019-nCoV_N1 IVT crRNA	GTATAATACGACTCACTATAGG	ACCCCGCATTACGTTTGGTGGACCGTTTTAG TCCCCTTCGTTTTTGGGGTAGTCTAAATCCCT ATAGTGAGTCGTATTATAC
3b	2019-nCoV_N2 IVT crRNA	GTATAATACGACTCACTATAGG	CACAATTTGCCCCCAGCGCTTCAGCGGTTTT AGTCCCCTTCGTTTTTGGGGTAGTCTAAATC CCTATAGTGAGTCGTATTATAC
3b	2019-nCoV_N3 IVT crRNA	GTATAATACGACTCACTATAGG	GATCACATTGGCACCCGCAATCCTGCTGTTT TAGTCCCCTTCGTTTTTGGGGTAGTCTAAAT CCCTATAGTGAGTCGTATTATAC
5d	LwCas13a orf1ab IVT crRNA	GTATAATACGACTCACTATAGG	ATAGTTTAAAAATTACAGAAGAGGTTGGGT TTTAGTCCCCTTCGTTTTTGGGGTAGTCTAAA TCCCTATAGTGAGTCGTATTATAC
3b	2019-nCoV_N1 PCR amplification T7	GAAATTAATACGACTCACTATAGGGACCCCAAA ATCAGCGAAAT	TCTGGTTACTGCCAGTTGAATCTG
3b	2019-nCoV_N2 PCR amplification T7	GAAATTAATACGACTCACTATAGGGTTACAAACA TTGGCCGCAAA	GCGCGACATTCCGAAGAA
3b	2019-nCoV_N3 PCR amplification T7	GAAATTAATACGACTCACTATAGGGAGCCTTGA ATACACCAAAA	TGTAGCACGATTGCAGCATTG
5d	Orf1ab RPA amplification T7[1]	GAAATTAATACGACTCACTATAGGGCGAAGTTG TAGGAGACATTATACTTAAACC	TAGTAAGACTAGAATTGTC TACATAAGCAGC

Supplementary Table 5: Primer and Probe Sequences for LAMP Reactions

Figure	Name	Sequence	Final Concentration
3d, 5e	GeneN-A-F3	TGGCTACTACCGAAGAGCT	1.6 µM
	GeneN-A-B3	TGCAGCATTGTTAGCAGGAT	1.6 µM
	GeneN-A-FIP	TCTGGCCCAGTTCCTAGGTAGTCCAGACGAATTCGTGGTGG	0.2 µM
	GeneN-A-BIP	AGACGGCATCATATGGGTTGCACGGGTGCCAATGTGATCT	0.2 µM
	GeneN-A-LF	GGACTGAGATCTTTCATTTTACCGT	0.4 µM
	GeneN-A-LB	ACTGAGGGAGCCTTGAATACA	0.4 µM

Primer sequences from Zhang *et al.*[2]

References

1. Metsky, H. C., Freije, C. A., Kosoko-Thoroddsen, T.-S. F., Sabeti, P. C. & Myhrvold, C. CRISPR-based COVID-19 surveillance using a genomically-comprehensive machine learning approach. *bioRxiv* 2020.02.26.967026 (2020) doi:10.1101/2020.02.26.967026.

2. Zhang, Y. *et al.* Rapid Molecular Detection of SARS-CoV-2 (COVID-19) Virus RNA Using Colorimetric LAMP. *medRxiv* **2**, 2020.02.26.20028373 (2020).
