# Supplementary figures and images for: A role for Biofoundries in rapid development and validation of automated SARS-CoV-2 clinical diagnostics
Source: Nat Commun. 2020 Sep 8;11:4464. doi: 10.1038/s41467-020-18130-3 (PMC7479142; doi:10.1038/s41467-020-18130-3)

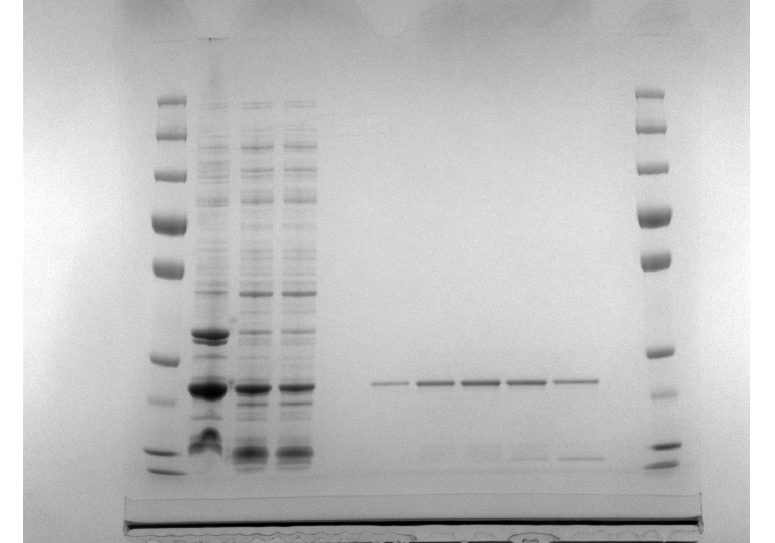

Supplement: Supplementary file 4 — Source Data [file 41467_2020_18130_MOESM4_ESM.zip › Data/MS2_VLP_Purification_SDS_PAGE.png]
